# 881. Long-term Weight Gain After Initiating Combination Antiretroviral Therapy in Treatment-naïve Asian People Living with HIV

**DOI:** 10.1093/ofid/ofab466.1076

**Published:** 2021-12-04

**Authors:** Naokatsu Ando, Takeshi Nishijima, Daisuke Mizushima, Yosuke Inaba, Yohei Kawasaki, Yoshimi Kikuchi, Hiroyuki Gatanaga, Shinichi Oka

**Affiliations:** 1 National Center for Global Health and Medicine, Shinjuku-ku, Tokyo, Japan; 2 Chiba University, Chuo-ku, Chiba, Japan

## Abstract

**Background:**

Weight gain after the initiation of antiretroviral therapy (ART) is becoming a major clinical issue in treatment-naïve people living with human immunodeficiency virus (PLWH). However, limited data exist for the Asian populations. We aimed to investigate changes in weight after the initiation of ART therapy in treatment-naïve Asian patients.

**Methods:**

We evaluated adult, treatment-naïve Asian PLWH who started integrase strand transfer inhibitor (INSTI)-, protease inhibitor (PI)-, or nonnucleoside reverse transcriptase inhibitor (NNRTI)-based ART at AIDS Clinical Center, Tokyo, between January 2005 and February 2019. They were followed up until October 2019. Multivariate linear mixed-effects models were used to generate marginal predictions of weight over time. Predicted weight by ART class (INSTI, PI, and NNRTI), each key drug (dolutegravir [DTG], elvitegravir [EVG], raltegravir [RAL], and darunavir [DRV]), and each key drug with or without the use of tenofovir alafenamide (TAF)/emtricitabine (FTC) was reported at 3-month intervals until censoring or 5 years.

**Results:**

Among the 1,579 study patients, 610 (38.6%), 929 (58.8%), and 40 (2.5%) started INSTI-, PI-, and NNRTI-based ART. After 5 years, PLWH who initiated DTG- (5.3 kg), DRV- (4.0 kg), and EVG-based treatment (4.6 kg) gained more weight than those who initiated RAL-based treatment (1.8 kg). PLWH who initiated DTG plus TAF/FTC (6.7 kg) gained the largest weight.

**Conclusion:**

In the Asian PLWH population, ART-associated weight gain continues to increase for 5 years after treatment initiation. DTG plus TAF/FTC was associated with the largest weight gain.

**Disclosures:**

**All Authors**: No reported disclosures

